# Supplementary material for: In vivo elongation of thin filaments results in heart failure
Source: PLoS One. 2020 Jan 3;15(1):e0226138. doi: 10.1371/journal.pone.0226138 (PMC6941805; doi:10.1371/journal.pone.0226138)
Supplement: S1 Fig — (DOCX) [file pone.0226138.s002.docx]

**
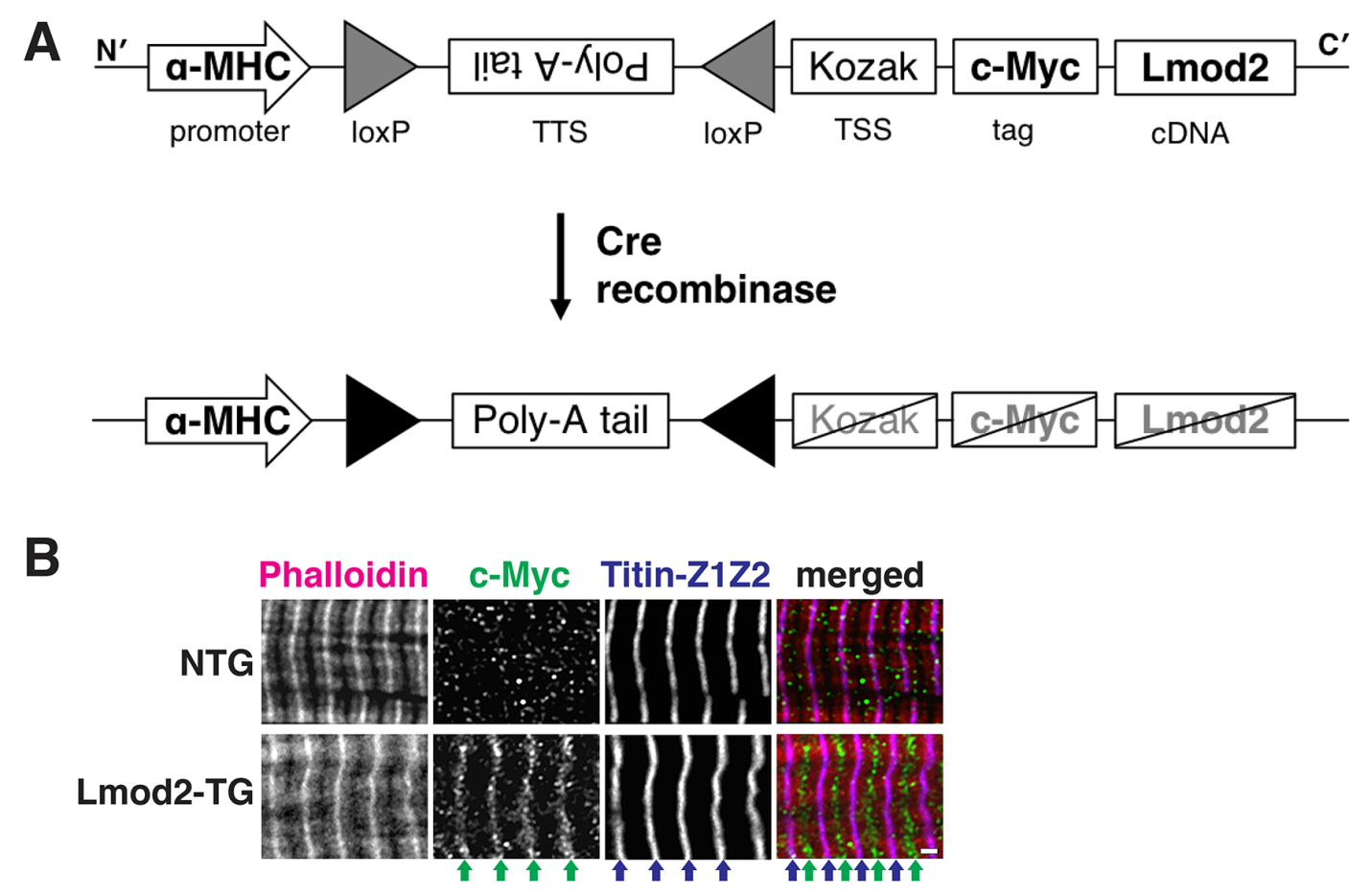
**

**Supporting Figure *S1*. Schematic of experimental design used to generate the Lmod2-TG transgenic mouse strain.**

**(A)** Alpha-myosin heavy chain (α-MHC) promoter ensures cardiac tissue-specific expression of an N-terminally c-Myc-tagged *Lmod2* transgene. Expressing *Cre*-recombinase via adeno-associated viral transduction (AAV_Cre) silences transgene expression. Recombination, following delivery of AAV_Cre, flips a reversed transcription termination sequence (TTS; Poly-A tail) encased between the two loxP sites and efficiently prevents the overexpression of Lmod2 transgene. TSS – eukaryotic translation start (Kozak) sequence. **(B)** Neonatal cardiomyocytes isolated from non-transgenic (NTG) control and Lmod2 transgene-positive (Lmod2-TG) hearts were fixed at four days after plating and subjected to immunofluorescent staining. Fluorescent signals: phalloidin (*red in merged*; filamentous actin), c-Myc (*green in merged*); Titin-Z1Z2 (*blue in merged* and *dark blue arrows*; Z-discs/thin filament barbed ends). Only the background level of c-Myc signal is detected in NTG, whereas a striated pattern at thin filament pointed ends (*green arrows*) is detected in Lmod2-TG cells. Scale bar = 1 μm.
