# Supplementary material for: In vivo elongation of thin filaments results in heart failure
Source: PLoS One. 2020 Jan 3;15(1):e0226138. doi: 10.1371/journal.pone.0226138 (PMC6941805; doi:10.1371/journal.pone.0226138)
Supplement: S2 Fig — (DOCX) [file pone.0226138.s003.docx]

**
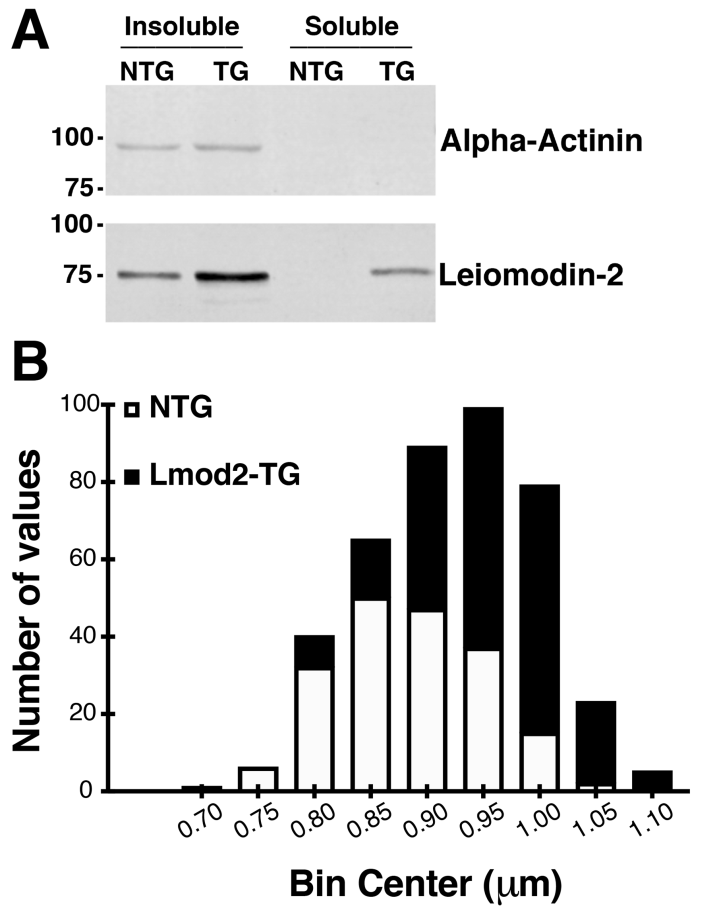
**

**Supporting Figure *S2*. Higher levels of Lmod2 expression are detected in both soluble and insoluble fractions of Lmod2-TG hearts, which have consistently longer thin filaments.**

**(A)** Representative immunoblots of insoluble (assembled) and soluble fractions extracted from left ventricular tissue of P7 NTG and Lmod2-TG mice are probed with anti-Lmod2 (*bottom*; ~80-kDa) and anti-sarcomeric α-actinin (*top*; ~100-kDa) antibodies. Sarcomeric α-actinin, as well as the endogenous expression level of Lmod2 in NTG, are undetectable in soluble protein fractions. N = 4 each. **(B)** Frequency distribution histogram of thin filament lengths at P7 show that those of Lmod2-TG (*black*) are consistently longer than NTG (*white*). N = 190-217 measurements from 6 mice each.
