# Supplementary material for: In vivo elongation of thin filaments results in heart failure
Source: PLoS One. 2020 Jan 3;15(1):e0226138. doi: 10.1371/journal.pone.0226138 (PMC6941805; doi:10.1371/journal.pone.0226138)
Supplement: S3 Fig — (DOCX) [file pone.0226138.s004.docx]

**
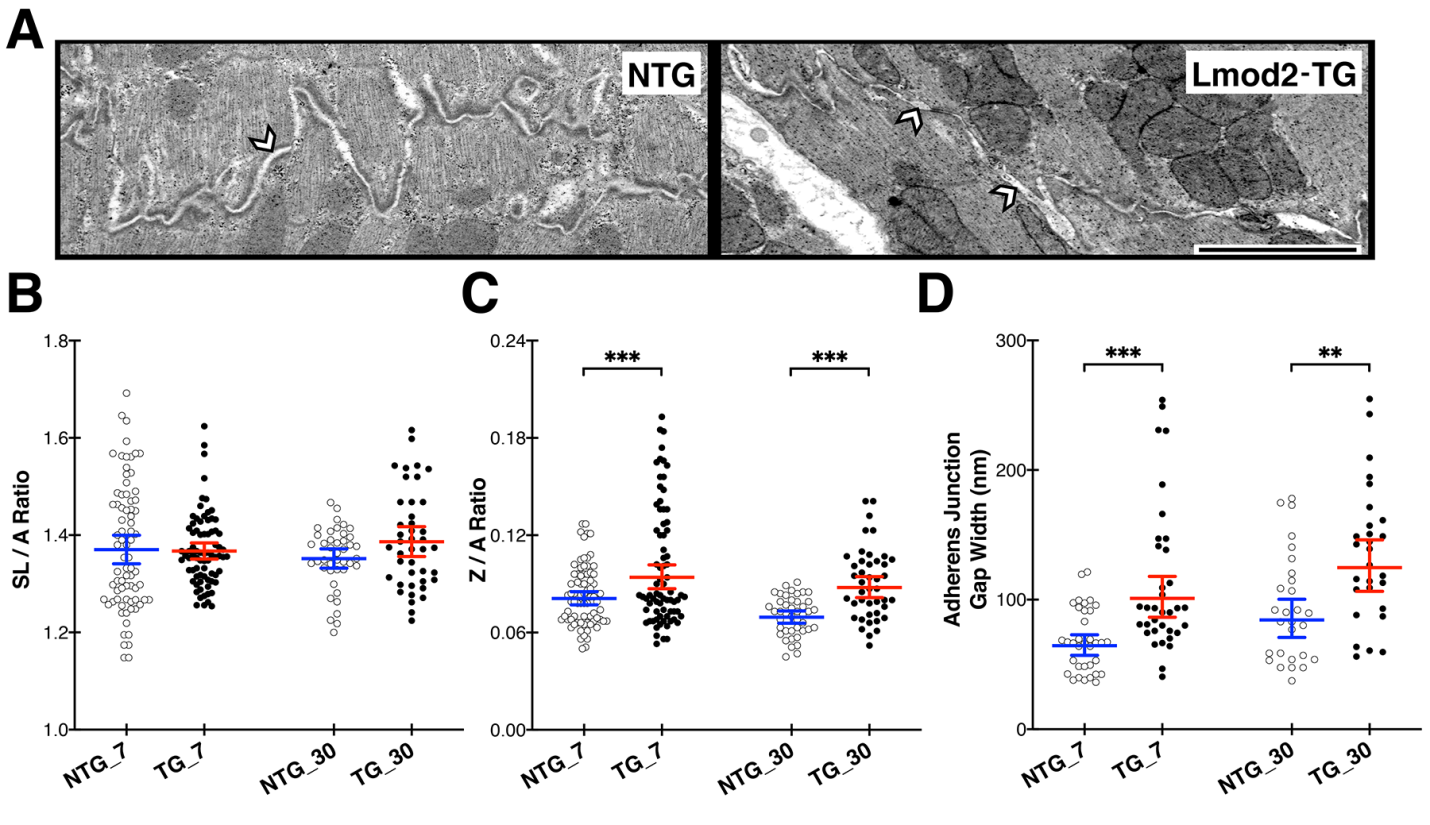
**

**Supporting Figure *S3*. Quantitative analysis of TEM indicates that Lmod2-TG hearts possess significantly wider Z-discs and adherens junctions, hallmarks of cardiac myopathy.**

**(A)** Intercalated discs (*chevrons*) of P30 Lmod2-TG heart are distinctively disrupted with wider gaps between the major composite junctional complexes when compared to NTG (representative images from n = 3 per genotype; scale bar = 2 μM). Quantitative measurements of TEM samples: **(B)** Sarcomere length adjusted to corresponding A-band width (SL/A ratio); **(C)** Z-disc width adjusted to corresponding A-band width (Z/A ratio); **(D)** Gap width of adherens junction of the intercalated discs. Measurement of P7 and P30 samples were denoted as ‘_7’ and ‘_30’ respectively. N = 42-76 measurements (B-C) and 28-35 measurements (D) from 3 animals each; Error bar = Mean ± 95% Confidence Interval; Blue error bar and line: NTG; Red error bar and line: Lmod2-TG (TG); ordinary one-way *ANOVA*, *Sidak* test.
