# Supplementary material for: In vivo elongation of thin filaments results in heart failure
Source: PLoS One. 2020 Jan 3;15(1):e0226138. doi: 10.1371/journal.pone.0226138 (PMC6941805; doi:10.1371/journal.pone.0226138)
Supplement: S4 Fig — (DOCX) [file pone.0226138.s005.docx]

**
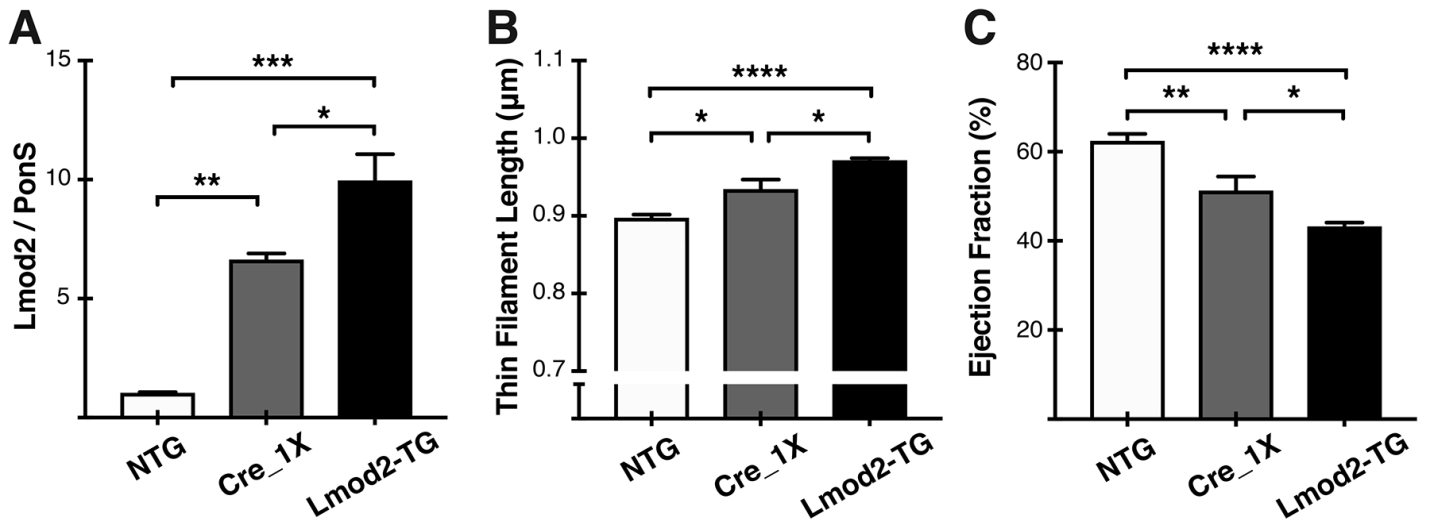
**

**Supporting Figure *S4*. Incomplete silencing of *Lmod2* transgene expression results in partial prevention (rescue) of aberrant phenotypes in Lmod2-TG animals.**

Lmod2-TG pups injected with one-tenth of the *Cre*-recombinase used in Figure 6 (*Cre_1X; gray bars*) still have higher Lmod2 protein levels **(A)**, longer thin filaments **(B)** and reduced percent ejection fraction **(C)** when compared to NTG, while having significantly lower Lmod2 protein levels, shorter thin filaments and higher percent ejection fraction when compared to Lmod2-TG animals. N = 4-6; Error bar = SEM; ordinary one-way *ANOVA*, *Tukey* test; ** P<0.05; ** P<0.01; *** P<0.001; **** P<0.0001*.
