# Supplementary material for: In vivo elongation of thin filaments results in heart failure
Source: PLoS One. 2020 Jan 3;15(1):e0226138. doi: 10.1371/journal.pone.0226138 (PMC6941805; doi:10.1371/journal.pone.0226138)
Supplement: S5 Fig — (DOCX) [file pone.0226138.s006.docx]

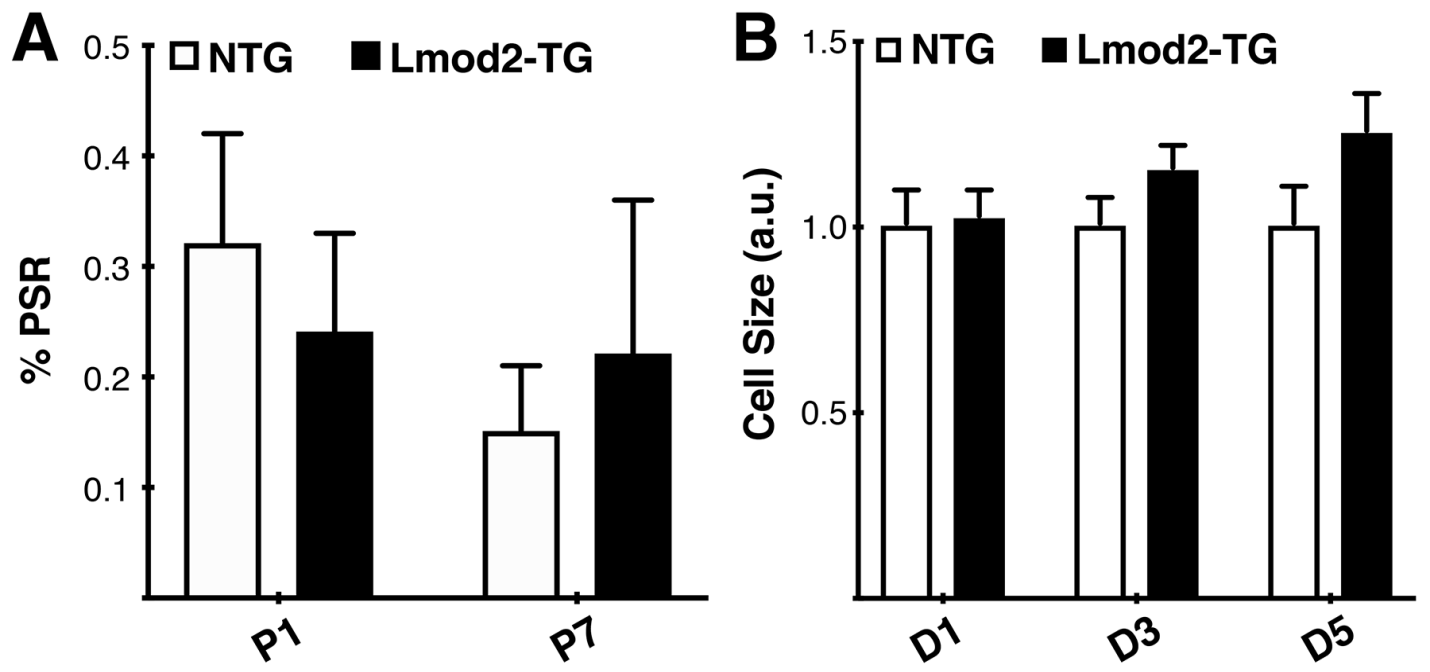


**Supporting Figure *S5*. The levels of interstitial fibrosis in Lmod2-TG hearts in younger mice, and cardiomyocyte size in Lmod2-TG neonatal cardiomyocytes are not significantly different from those of NTG.**

**(A)** Quantification of Picrosirius Red-stained (%PSR) ventricular tissues of P1 and P7 mice indicates no significant differences in levels of interstitial fibrosis (i.e., collagenous deposits) between Lmod2-TG (*black*) and NTG (*white*) (n = 4-10; Error bar = SEM; *two-tailed unpaired Welch’s t-test*). **(B)** To determine cell size surface areas of neonatal cardiomyocytes isolated from P2 Lmod2-TG (*black*) and NTG (*white*) were measured using ImageJ, and analyzed at day 1 (D1), D3 and D5 post-plating. Although Lmod2-TG cell size is trending to be larger at D5, no statistically significant difference was detected (n = 30 cells per genotype per time point; 2 neonatal cardiomyocyte cultures; Error bar = SEM; *two-tailed unpaired Welch’s t-test*).
