# Supplementary material for: In vivo elongation of thin filaments results in heart failure
Source: PLoS One. 2020 Jan 3;15(1):e0226138. doi: 10.1371/journal.pone.0226138 (PMC6941805; doi:10.1371/journal.pone.0226138)
Supplement: S1 Table — (DOCX) [file pone.0226138.s007.docx]

**Supporting Table *S1.* Comparison of RNA transcript levels between NTG and Lmod2-TG.**

Analysis of the 100-bp paired-end RNA-sequencing data generated using mRNA extracted from left ventricular tissue of P7 mice. FDR – False discovery rate. Significance of differentially expressed genes was determined when Log2 ≤ -2 (down-regulated in Lmod2-TG) and Log2 ≥ 2 (up-regulated in Lmod2-TG). Fold difference was determined by dividing fragments per kilobase of exon per million fragments mapped (FPKM) value of a gene-of-interest in Lmod2-TG sample by the respective FPKM value of NTG. Data are sorted in the decreasing order of fold difference within each group (refer to ‘NOTE’ column). N = 3 biological replicates per genotype.
