# Supplementary material for: In vivo elongation of thin filaments results in heart failure
Source: PLoS One. 2020 Jan 3;15(1):e0226138. doi: 10.1371/journal.pone.0226138 (PMC6941805; doi:10.1371/journal.pone.0226138)
Supplement: S2 Table — (DOCX) [file pone.0226138.s008.docx]

**Supporting Table *S2.* Morphometric analyses of NTG and Lmod2-TG.**

Morphometric analyses of non-transgenic control (NTG) and Lmod2-TG (TG) mice were carried out at post-natal day (P) 1, 7, 15, 30 and 60. BW = body weight (g); TL = tibia length (mm; not applicable at P1); HW = heart weight (mg); LW = lung weight (mg); HW/BW and LW/BW in mg/g; HW/TL and LW/TL in mg/mm. *Unpaired two-tailed t-test*. ** P<0.05; ** P<0.01; **** P<0.0001.*
