# Supplementary material for: In vivo elongation of thin filaments results in heart failure
Source: PLoS One. 2020 Jan 3;15(1):e0226138. doi: 10.1371/journal.pone.0226138 (PMC6941805; doi:10.1371/journal.pone.0226138)
Supplement: S3 Table — (DOCX) [file pone.0226138.s009.docx]

**Supporting Table *S3.* Left ventricular (LV) echocardiography analyses of NTG and Lmod2-TG mice via M-mode.**

LV M-mode echocardiography of non-transgenic control (NTG) and Lmod2-TG (TG) littermates were tracked every week: post-natal week 1 (P7), week 2 (P14), week 3 (P21), week 4 (P28) and week 8 (P56-60). N = 6-8 mice per genotype (equal numbers of males and females) from 3 litters. Notes: (1) echocardiography data gathered without using anesthesia; (2) no significant difference observed between male and females; (3) echocardiography data gathered using anesthesia; and (4) significant difference observed between males and females (primarily due to females having smaller-sized hearts). Because of the unusual geometry of Lmod2-TG hearts (Fig. 2 *A-B*), values associated with the systolic phase of cardiac cycle were not included in data interpretation (refer to Fig.6*C* and Table *S6* for comprehensive load-independent data analysis). Diastolic values (_d) of internal diameter (ID), posterior wall thickness (PW), anterior wall thickness (AW), and eccentricity (a measure of LV hypertrophy), heart rate (HR), percent ejection fraction (%EF) and percent fractional shortening (%FS) are reported here. Eccentricity was calculated as end-diastolic diameter (LVEDD; ID_d) divided by the wall thickness (WT; average of PW_d and AW_d). *Two-tailed unpaired t-test* (P7 – P28); two-way *ANOVA* with *Tukey* test (P60). ** P<0.05; ** P<0.01.* Asterisk (*) for comparison between Lmod2-TG and NTG. Caret (^) for comparison between Lmod2-TG and Lmod2-cre.
