# Supplementary material for: In vivo elongation of thin filaments results in heart failure
Source: PLoS One. 2020 Jan 3;15(1):e0226138. doi: 10.1371/journal.pone.0226138 (PMC6941805; doi:10.1371/journal.pone.0226138)
Supplement: S4 Table — (DOCX) [file pone.0226138.s010.docx]

**Supporting Table *S4*. Summary of single cardiac fiber mechanics study**.

Single-fiber mechanic experiments were performed using trabeculae isolated from P15 and P60 mice. N = 1 fiber per animal from 7-9 mice. *Brown-Forsythe and Welch ANOVA*; ** P<0.05; ** P<0.01; *** P<0.001.*
