# Supplementary material for: In vivo elongation of thin filaments results in heart failure
Source: PLoS One. 2020 Jan 3;15(1):e0226138. doi: 10.1371/journal.pone.0226138 (PMC6941805; doi:10.1371/journal.pone.0226138)
Supplement: S5 Table — (DOCX) [file pone.0226138.s011.docx]

**Supporting Table *S5*. Cardiac reserve in NTG and Lmod2-TG mice.**

LV M-mode echocardiography measurements were obtained before (*baseline*) and after (*post-dobutamine*) the administration of dobutamine, the beta1-agonist. Measurements representing systolic performance, such as heart rate, percent ejection fraction and percent fractional shortening, are reported here. All values are significantly improved in NTG mice, whereas in Lmod2-TG mice only the heart rate is improved (by ~27%) after dobutamine treatment. N = 4 (2M, 2F) each; *two-tailed paired parametric t-test*; ** P<0.05*; *** P<0.01*.
