# Supplementary material for: In vivo elongation of thin filaments results in heart failure
Source: PLoS One. 2020 Jan 3;15(1):e0226138. doi: 10.1371/journal.pone.0226138 (PMC6941805; doi:10.1371/journal.pone.0226138)
Supplement: S6 Table — (DOCX) [file pone.0226138.s012.docx]

**Supporting Table *S6*. Pressure-Volume Loop (PV-Loop) analysis.**

PV-Loop analysis indicates that Lmod2-TG mice have both systolic- and diastolic dysfunctions. Ees = end-systolic elastance; Tau = time constant of isovolumic relaxation calculated using various (Glantz, Mirsky and Weiss) methods. *Two-tailed unpaired t-test* (initially between NTG and Lmod2-TG); two-way *ANOVA* with *Tukey* test (among all groups). Asterisk (*) for comparison between NTG and Lmod2-TG, caret (^) for comparison between Lmod2-TG and Lmod2-cre, and pound (#) for comparison between NTG and Lmod2-cre. ** P<0.05; ** P<0.01; *** P<0.001; **** P<0.0001.*
