# Supplementary material for: In vivo elongation of thin filaments results in heart failure
Source: PLoS One. 2020 Jan 3;15(1):e0226138. doi: 10.1371/journal.pone.0226138 (PMC6941805; doi:10.1371/journal.pone.0226138)
Supplement: Attachment — Submitted filename: PONE-D-19-23641 Mi-Mi et al.docx [file pone.0226138.s013.docx]

Review of PONE-D-19-23641 submitted by Mi-Mi et al.

Title of MS: In vivo elongation o thin filaments results in heart failure.

This is a beautifully written, well documented and carefully deduced conclusions on the effects of increasing the amount of a thin filament pointed end associated protein leiomodin-2 in mouse models. Increasing the level of this protein resulted in a 10 to sometimes 26% longer thin filaments. Examination of the effects of raising the intracellular levels of lmod2 also lead to effects on the Z-Bands and intercalated discs in the mouse hearts.. The sarcomeres from muscle expressing this higher level of Lmod2 resulted in a loss of upto 50% loss of contractile forces resulting in cardiac failure. It is very impressive that their experiments in which they lower the high levels on Lmod2 lead to a reversal of the effects of the high levels of Lmod2. The authors have previously demonstrated that lowering the amount of Lmod in the cardiomyocytes lead to shorter filaments, and also cardiac defects. The combination of these two reports demonstrates the importance of Lmod in the health of the heart. This manuscript is a first-rate contribution to our understanding of the role of Lmod2 in myofibril assembly, and how changing the normal levels of Lmod2 can lead to cardiac failure. This manuscript should be published without changes.
