# Supplementary material for: In vivo elongation of thin filaments results in heart failure
Source: PLoS One. 2020 Jan 3;15(1):e0226138. doi: 10.1371/journal.pone.0226138 (PMC6941805; doi:10.1371/journal.pone.0226138)
Supplement: Attachment — Submitted filename: PONE-D-19-23641_Response to Reviewers.docx [file pone.0226138.s014.docx]

**RESPONSE TO REVIEWERS**

1. *“The authors should better highlight limitations of the study”*.

The only limitation of this study that we are aware of is that we are not able to study the mice homozygous for the Lmod2 transgene. This is addressed as follows:

*Heterozygous Lmod2 transgene-positive (Lmod2-TG) mice do not die prematurely and are able to breed when Lmod2-TG males are crossed with Lmod2 transgene-null (NTG) females. However, the majority of Lmod2-TG females do not survive through pregnancy, potentially due to volume-induced cardiac stress (n = 10; percent fatality = 60%), and homozygous Lmod2 transgenic pups do not survive past post-natal day 7 (n = 3 litters; percent fatality = 100%)*.

Thus, analysis of homozygous Lmod2 transgenic mice could not be included in this study. See page16, lines 313-318.

2. *“Moreover, it is important to measure capillary density, cardiomyocyte size and collagen content*.”

We are a bit confused about the question the Editor would like us to address with this request but, hopefully, we sufficiently addressed the request below. In short, we do not have any data that demonstrates statistically significant differences between Lmod2-TG and NTG mice, in terms of capillary density, cardiomyocyte size or collagen content *before Lmod2-TG hearts display cardiac disease remodeling* (**P30**; ***Fig. 2***). In comparison, cardiac thin filament lengths are already 3% longer at P1, and contractile force reduced by 47% at P15.

- **RE: Capillary density**

We deduce that this query arose from two possible scenarios: (1) increases in capillary density accompany hypertrophied hearts; or (2) decreases in capillary density give way to tissue hypoxia, cell death and interstitial fibrosis. Both scenarios could lead to contractile dysfunction and heart failure.

Note, #1 is unlikely a primary factor contributing to heart failure because we only observe cardiac hypertrophy (i.e., increased heart mass) in Lmod2-TG mice at P60 (**Supporting Table *S3***), when disease remodeling has already taken place.

As for #2, we found that: (i) levels of interstitial fibrosis in Lmod2-TG are only significantly elevated after P30 (*Fig. 2C*), and not at P1 and P7 as indicated by Picrosirius Red-staining and quantification (%PSR) (which is now added as a **Supporting Figure *S5A***; n = 4-10; error bars = SEM); and (ii) passive tension average (a criterion linked to fibrosis levels) between NTG and Lmod2-TG is not statistically significant at P15 (*Fig. 5C*).

It is also important to note that we do not detect changes in the levels of *Lmod1*, the leiomodin isoform predominantly expressed in smooth muscles, as well as other markers of pathological capillary densities, in our Lmod2-TG model (**Supporting Table *S1***). Together, these data suggest that changes in capillary density are unlikely a primary contributing factor to heart failure in the Lmod2-TG mice.

**Added: Supporting Figure *S5A***

**Updated: Supporting Table *S1***

- **RE: Cardiomyocyte size**

Surface areas of neonatal cardiomyocytes extracted from P2 Lmod2-TG and NTG littermates at day 1 (D1), day 3 (D3) and day 5 (D5) post-plating were measured using ImageJ. No statistically significant difference was detected at all time points examined. These data are now added as a **Supporting Figure *S5B*** (n = 30 each; error bars = SEM).

**Added: Supporting Figure *S5B***

- **RE: Collagen content**

We used Picrosirius-Red staining to detect collagenous deposits (see above). No significant difference between NTG and Lmod2-TG in the percent Picrosirius Red-stained (%PSR) P1 and P7 LV sections was detected (n = 4-10). In addition, we detected no significant difference in collagen gene expressions in our RNA-Seq between P7 NTG and Lmod2-TG. These data have been added to **Supporting Table** ***S1*** (*P < 0.05*; n = 3 per genotype).

**Updated: Supporting Table *S1***

3. *“Please ensure that your manuscript meets PLOS ONE's style requirements, including those for file naming*.”

This has been addressed.

4. *“PLOS ONE now requires that authors provide the original uncropped and unadjusted images underlying all blot or gel results reported in a submission’s figures or Supporting Information files.”*

This requirement has been fulfilled. See below.

**Main Figure *1B***

Original picture (1 of 3). Note: This nitrocellulose strip was cut from the large blot displayed in 3/3 below; Leiomodin-2; ~80.kDa; *red-boxed region*

Original picture (2 of 3). Note: This nitrocellulose strip was cut from the large blot displayed in 3/3 below; c-Myc; ~80.kDa; *green-boxed region*

Original picture (3 of 3). Ponceau-S stained membrane; *black-boxed region*

– 250.kDa

– 130.kDa

– 100.kDa

– 70.kDa

– 55.kDa

– 35.kDa

**Supporting Figure *S2A***

Original image (1 of 3). The whole blot probed with mouse monoclonal anti-sarcomeric alpha-actinin; ~100.kDa; *green-boxed region*

Original image (2 of 3). The same blot as above probed with rabbit poly-clonal anti-leiomodin-2; ~80.kDa; *red-boxed region*

Original image (3 of 3). Ponceau-S stained membrane; *black-boxed region*

1 – 250.kDa

2 – 150.kDa

3 – 100.kDa

4 – 75.kDa

5 – 50.kDa

All Extracted Proteins

(not included)

Insoluble Proteins

Soluble Proteins
